# Performance Study of Wearable Thermoelectric Cooler with Phase-Change Composite Heat Sink

**DOI:** 10.3390/ma18071576

**Published:** 2025-03-31

**Authors:** Zhanglong Xia, Wei Cao, Xiaolong Sun, Qianfeng Ding, Zheng Zhu, Wenjie Zhou, Sijia Yan, Yue Hou, Ziyu Wang

**Affiliations:** The Institute of Technological Sciences, Wuhan University, Wuhan 430072, China; xiazhanglong@whu.edu.cn (Z.X.); wei_cao@whu.edu.cn (W.C.); xiaolongsun@whu.edu.cn (X.S.); qfding@whu.edu.cn (Q.D.); zhengzhu@whu.edu.cn (Z.Z.); wenjiezhou@whu.edu.cn (W.Z.); yansijia@whu.edu.cn (S.Y.)

**Keywords:** wearable thermoelectric cooler, personal thermal management, phase-change composite material, simulation analysis

## Abstract

Based on existing studies, we identified that the heat sinks used in wearable thermoelectric coolers (WTECs) are predominantly bulky, which limits their practicality and comfort. To address this issue, we propose the use of phase-change composite materials (PCCMs) due to their inherent flexibility and thermal properties. Through comprehensive theoretical analysis, numerical simulations, and experimental validation, we successfully optimized the design of a WTEC.

## 1. Introduction

Facing the reality of existing extremely hot weather, personal thermal management technology, which enhances the overall thermal comfort by cooling the specific area, is garnering increasing attention [1,2,3,4,5]. Within multiple cooling performance, thermoelectric devices hold significant potential due to their temperature-controllable cooling ability [6,7,8,9,10]. However, the devices’ cooling durations are highly restricted by the poor heat dissipation on their hot ends [11,12,13,14,15,16]. Previous studies have shown that adding heat sinks or improving heat ventilation (or heat exchange) can both enhance cooling performance [17,18,19,20,21,22].

The sophisticated manufacturing technology employed by the thermoelectric device will also serve as a cornerstone for enhancing its operational flexibility and resilience [23,24]. Hou et al. introduced ultra-flexible fabric-based thermoelectric generators, incorporating conductive fabric electrodes and elastic fabric substrates [24]. Combined with serpentine-structured fabric electrodes, the device exhibits exceptional structural integrity and flexibility. At the same time, conductive polymers are highly suitable for use in wearable thermoelectric devices in low-temperature regions [25,26], as their flexibility and biocompatibility provide significant advantages over inorganic materials. To tackle the issue of low carrier mobility in conducting polymers, Gharahcheshmeh et al. investigated the nanostructures of poly(3,4-ethylenedioxythiophene) films fabricated via oxidative chemical vapor deposition at various deposition temperatures [26], and finally they achieved a substantial improvement in carrier mobility and conductivity. Indeed, efficient heat dissipation remains a critical consideration for both fabric-based devices and those utilizing conductive polymers.

He et al. investigated the performance of plate-fin heat sinks using rectangular and V-shaped fins under lateral and vertical convection conditions and in multiple arrangements. Their results showed that rectangular fins arranged in double rows exhibited the highest heat dissipation capacity on the hot end of the thermoelectric cooler under vertical jet conditions [18]. Mark Baldry et al. developed an optimized natural convection heat sink through iterative numerical simulations for a small thermoelectric cooler (TEC) module, achieving a steady-state reference temperature 11.7 °C lower than a conventional design [27]. Liu et al. demonstrated the potential of integrating heat pipes into thermoelectric cooling systems for electronic device applications [28]. Y. Lyu et al. developed a battery thermal management system combining thermoelectric, forced air, and liquid cooling methods, and the experimental results showed good cooling effectiveness and reasonable power consumption [29]. However, the application of the aforementioned heat dissipation methods in wearable thermal management is not applicable due to their limitations, including bulky size and lack of flexibility.

To address the cooling issue and at the same time provide a wearable-compatible solution for TEC heat dissipation, phase-change material (PCM) becomes a perfect solution. In multiple application scenarios, thermal management based on PCM has been reported [30,31,32,33,34,35,36,37,38,39]. Xiang et al. prepared polyurethane rigid foam composites which are made via in situ foaming the polyurethane prepolymer blended with microencapsulated phase-change material [30]. Huang et al. prepared a novel anti-leakage and anti-vibration thermally induced flexible phase-change composite material which utilized Ethylene–Propylene–Diene Monomer to increase the crosslinking of styrene–butadienestyrene block copolymer so as to improve the adsorption of paraffin (PA) [31]. Rohan Bhuiya et al. utilized ANSYS 18.1 to analyze the impact of phase-change materials on heat dissipation, specifically investigating various shapes of heat sinks integrated with these materials on thermoelectric coolers. Their findings indicated that the use of phase-change materials significantly lowered the hot-side temperature of the thermoelectric cooler compared to scenarios without such materials, highlighting their potential in thermoelectric cooling system thermal management [32]. Wang et al. performed a numerical comparative study on heat sink modules transitioning between single-phase and solid–liquid phase, examining both solid and unconsolidated porous structures. Their findings clearly demonstrated that phase-transitioned materials can markedly decrease the cold surface temperature and enhance the transient coefficient of performance (COP) of TEC [33]. Since pure PCM is prone to leakage, the current research on thermoelectric devices with PCM is mainly focused on simulation.

At this stage, the phase-change composite materials (PCCMs) listed above are not flexible and they do not have a mature application when combined with TEC. Meanwhile, in this work, we developed anti-leakage PCCM to be used as a heat sink for flexible TECs. The base material, Epoxy Resin, can effectively encapsulate the PCM particles, thus avoiding leakage of liquid PCM. Due to the good flexibility of PCCM and the high heat capacity, the PCCM can be applied to flexible TEC as a heat dissipation layer for wearable use. Both COMSOL simulation (COMSOL Multiphysics 6.2) and experiments have found that higher PCM content and geometry (thickness) are more favorable to improve the cooling effect of wearable thermoelectric coolers (WTECs). Subsequent experiments demonstrated that the WTEC in this study could achieve a temperature drop of 2.5 °C on the human skin and sustain the cooling duration for up to 10 min.

In this work, to address the issue of bulky heat sinks in existing WTECs, we propose the use of flexible PCCMs for efficient heat dissipation. Through theoretical analysis, simulation studies, and experimental validation, we demonstrate that the integration of PCCM enables the WTEC to achieve a temperature reduction of 2.5 °C on human skin, offering a promising solution for enhanced thermal comfort.

## 2. Experimental Section

### 2.1. Materials and Reagents

The materials we use are all commercially available. The p-type Bi_0.5_Sb_1.5_Te_3_ and n-type Bi_2_Te_0.3_Sb_2.7_ thermoelectric legs with the size of 1.4 × 1.4 × 2.5 mm^3^ were purchased from Hubei Segre New Energy Technology, Company Ltd., Wuhan, China. Our thermoelectric leg material parameters are consistent with those used in another study [40]. The zT value of the p-type Bi_0.5_Sb_1.5_Te_3_ thermoelectric legs is 0.927, while the zT value of the n-type Bi_2_Te_0.3_Sb_2.7_ thermoelectric legs is 0.965, both measured at 303 K. The flexible printed circuit board (FPCB) was purchased from Jialichuang Technology Group, Company Ltd., Shenzhen, China. Epoxy Resin (Leini 105) was purchased from Huizhou Tinglan Technology Company, Huizhou, China, and PA (PCM-A-30) was purchased from Dongguan Zhangmutou Shengbang Plastic Raw Material Operation Department, Dongguan, China, as PCM.

### 2.2. Fabrication of PCCM and WTEC

The fabrication process of the PCCM is shown in Figure S2. Epoxy Resin A and B agents were added sequentially, mixed, and stirred at 50 °C for 10 min. PA was then added, mixed, and stirred at ratios of 5, 10, and 15 wt% for another 10 min. The mixture was then vacuumed for 5 min to remove any bubbles before being poured into a mold which measures 24 × 44 mm^2^. The mixture was then cured at 50 °C for 8 h, after which the PCCM was obtained.

The preparation detail for the WTEC is demonstrated in Figure S3. The overall FPCB size is 24 × 44 mm^2^. First, the FPCB was placed on an unheated heating plate and low-temperature solder paste [Sn_42_Bi_58_ (melting point is 138 °C)] was loaded onto the mask and swiftly printed onto the substrate electrode with a squeegee. Second, the p-type and n-type thermoelectric legs were deposited onto the bottom electrode of the FPCB. Next, the whole sample was heated up to 200 °C for solder bonding and the soldering time was 5 min. Subsequently, the top electrode was welded using the same technological process.

### 2.3. Testing Instruments and Methods

Pure Epoxy Resin and our PCCM underwent differential scanning calorimetry (DSC) experiments (NETZSCH, Munich, Germany) to assess their heat absorption and release capabilities. The DSC experiments were set to start at 18 °C and end at 55 °C, while the ramp-up and ramp-down rates were all 3 °C/min. All samples were tested in a nitrogen (N_2_) atmosphere. The optical photos of Pure Epoxy Resin and PCCM were characterized by OLYMPUS (Center Valley, PA, USA). The scanning electron microscope (SEM) images of p-type and n-type thermoelectric legs were characterized by Zeiss SIGMA (Oberkochen, Germany). The Fourier transform infrared spectrometer (FT-IR) curves of pure PA, pure Epoxy Resin, and PCCM were characterized with a Thermo Fisher Scientific (Waltham, MA, USA) Nicolet iS20, and the scanning range was 400–4000 cm^−1^. The bending and tensile properties were evaluated using a flexible electronic tester (Prtronic FT2000, Shanghai Mifang Electronic Technology, Company Ltd., Shanghai, China). The skin temperature before and after cooling was recorded using a thermal infrared imager (FLUKE Ti480 PRO, FLUKE, Norwich, UK). At the same time, a thermocouple was placed at the interface between the skin and the cold end of the WTEC to measure the temperature. The internal resistance of the device was measured by using an electrochemical analyzer (CHI650e, CH Instruments, Inc., Bee Cave, TX, USA). The data tested are the average values of at least three measurements.

## 3. Results and Discussion

### 3.1. Theoretical Model

**Figure 1:**
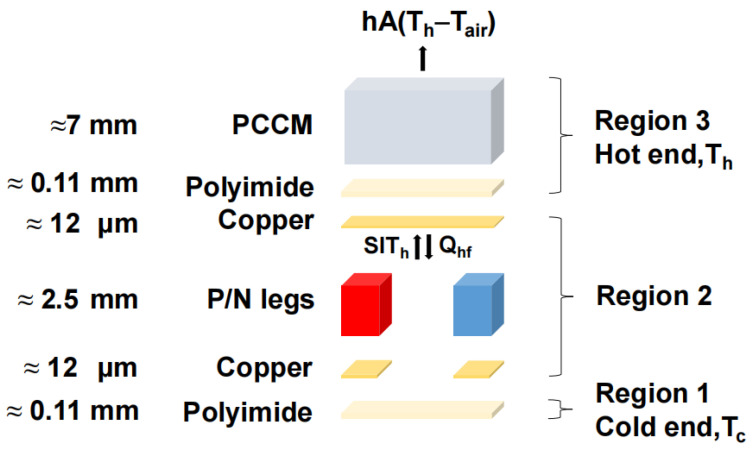
Schematic diagram of WTEC.

Figure 1 illustrates the schematic diagram of the WTEC, which includes p-type and n-type thermoelectric legs, copper connecting electrodes, and a polyimide substrate. The fundamental principle governing TEC devices is the Peltier effect.

We are very concerned with the cooling efficiency, or COP. The COP can be calculated using the following equation [34,35]:

(1) $$COP=\frac{T_{c}}{T_{h}-T_{c}}\frac{\sqrt{1+zT_{m}}-\frac{T_{h}}{T_{c}}}{1+\sqrt{1+zT_{m}}}$$

In this equation, $T_{c}$ represents the temperature at the cold end, $T_{h}$ represents the temperature at the hot end, z is the figure of merit of thermoelectric material, and $T_{m}$ is the average temperature between the cold end and the hot end. In this work, the transient response of the TEC is our primary focus. For the TEC depicted in the diagram, the energy equation for the hot end is as follows [34]:

(2) $$\left(M_{hpi}C_{hpi}+M_{PCCM}C_{PCCM})\frac{\mathrm{d}T_{h}}{\mathrm{d}t}=SIT_{h}-Q_{hf}-hA(T_{h}-T_{air}\right)$$

Here, $M_{hpi}$ represents the mass of polyimide at the hot end, $C_{hpi}$ denotes the specific heat capacity of polyimide at the hot end, $M_{PCCM}$ is the mass of PCCM, $C_{PCCM}$ is the specific heat capacity of PCCM, $T_{h}$ represents the temperature at the hot end, S is the Seebeck coefficient of the thermoelectric legs, I is the applied current, $Q_{hf}$ denotes the heat transfer rate between the hot end polyimide and the thermoelectric legs, h is the heat transfer coefficient at the hot surface of the thermoelectric device, A is total heat transfer surface area of the PCCM, and $T_{air}$ represents the ambient temperature [41].

From Equation (2), it can be observed that increasing the mass $M_{PCCM}$ and specific heat capacity [36] $C_{PCCM}$ of the PCCM can significantly enhance the heat capacity at the hot end of TEC, thereby reducing the rate of hot end temperature recovery $\frac{\mathrm{d}T_{h}}{\mathrm{d}t}$. The most straightforward method to increase the mass $M_{PCCM}$ of the PCCM is to adjust its geometric parameters. Notably, increasing the thickness not only enhances the heat capacity at the hot end but also increases the heat dissipation area, both of which contribute positively to controlling the temperature at the hot end. According to the apparent heat capacity method, increasing the content of PCM can significantly boost the specific heat capacity of the composite material [42]. This indicates that a reasonable enhancement of the thickness and PCM content of the PCCM is beneficial for the thermal management of the hot end of the device.

### 3.2. Simulation Module and Results

#### 3.2.1. Phase-Change Module

In the subsequent experiments, we observed that the PCCM had excellent shape stability during the phase-change process, which means the appearance is almost unchanged during the phase-change process, so we simulated the solid–solid phase change as a solid–solid phase change, and at the same time, we investigated the phase change by the apparent heat capacity method [43]. The following equation controls the phase-change module [44]:

(3) $$\rho_{pccm}c_{p,pccm}\frac{\partial T}{\partial t}=\nabla \left(k_{pccm}\nabla_{T}\right)$$

where $\rho_{pccm}$ represents the equivalent density of the PCCM, $c_{p,pccm}$ represents the equivalent specific heat capacity, T represents the temperature, $k_{pccm}$ represents the thermal conductivity of the PCCM, and t represents the time.

The equivalent thermal conductivity, equivalent density, and equivalent heat capacity of the PCCM are related to the phase-change fraction $\theta \left(T\right)$ and can be used to calculate the heat transfer in the material during the phase-change process. The values $k_{pccm}$, $\rho_{pccm}$, and $C_{p,pccm}$ of the PCCM are determined as follows during the phase-change process, respectively:

(4) $$k_{pccm}=k_{s1}+\left(k_{s2}-k_{s1}\right)\theta \left(T\right)$$

(5) $$\rho_{pccm}=\frac{\rho_{s2}C_{p,s2}\theta \left(T\right)+\rho_{s1}C_{\rho ,s1}\left(1-\theta \left(T\right)\right)}{C_{p,s2}\theta \left(T\right)+\left(1-\theta \left(T\right)\right)C_{p,s1}}$$

(6) $$C_{p,pccm}=C_{p,s1}+\left(C_{p,s2}-C_{p,s1}\right)\theta (T)+∆H_{composite}\cdot D\left(T\right)$$

$k_{s1}$ and $k_{s2}$ represent the thermal conductivity of the PCM in the PCCM before and after melting, respectively; $\rho_{s1}$ and $\rho_{s2}$ represent the density of the PCM in the PCCM before and after melting, respectively; $C_{\rho ,s1}$ and $C_{p,s2}$ represent the constant-pressure heat capacity of the PCM in the PCCM before and after melting, respectively; $∆H_{composite}$ represents the latent heat of the PCCM value; $D\left(T\right)$ is a Gaussian function to account for the latent heat in the melting temperature range; and the phase transition fraction $\theta \left(T\right)$ is used to describe the phase transition of the PCCM, and according to the principle of phase transition, the value of $\theta \left(T\right)$ can also be expressed as the following equation.

(7) $$\theta \left(T\right)=\left\{\begin{array}{c} 0,T<T_{m}-\frac{∆T}{2}\\ \frac{T-T_{m}+∆T/2}{∆T},T_{m}-\frac{∆T}{2}<T<T_{m}+\frac{∆T}{2}\\ 1,T>T_{m}+\frac{∆T}{2} \end{array}\right.$$

$T_{m}$ represents the melting temperature of the PCCM and ∆T represents the melting temperature range of the PCCM. In Section 2, we mention that we choose PA as the PCM. The PA we chose has a melting point of 30 °C, which meets the standard that it will not melt at room temperature. Since the PCCM is a shape-stabilized material, as confirmed by subsequent experiments, we can study the PCCM as a whole [44].

The formula for calculating the latent heat of the composite materials is shown below:

(8) $$∆H_{composite}=w_{PA}\times {∆H}_{PA}$$

$∆H_{composite}$ is the latent heat value of the shape-stabilized PCCM [45,46,47], $w_{PA}$ is the mass fraction of the phase-change material to the PCCM, and ${∆H}_{PA}$ is the latent heat value of the phase-change material.

In the simulations, the selected PA contents are 5 wt%, 10 wt%, 15 wt%, and 20 wt%, respectively.

#### 3.2.2. Thermoelectric Module

The non-steady-state energy equation for a p/n-type semiconductor column can be described as follows:

(9) $$\left(\rho_{p/n}C_{p/n}\right)\frac{\partial T}{\partial t}=\nabla \left(k_{p/n}\nabla T\right)+\frac{\vec{J^{2}}}{\sigma_{p/n}(T)}-\nabla S_{p/n}\vec{J}T_{p/n}$$

Here, $\rho_{p/n}$ denotes the equivalent density of the thermoelectric material, $C_{p/n}$ denotes the specific heat capacity of the thermoelectric material, T represents the temperature, t represents the time, $k_{p/n}$ denotes the thermal conductivity of the thermoelectric material, $\sigma_{p/n}(T)$ represents the electrical conductivity of the thermoelectric material, $T_{p/n}$ represents the temperature of the thermoelectric material, and $S_{p/n}$ denotes the Seebeck coefficient.

(10) $$\left(\rho_{cop}C_{cop}\right)\frac{\partial T}{\partial t}=\nabla \left(k_{cop}\nabla T\right)+\frac{\vec{J^{2}}}{\sigma_{cop}}$$

(11) $$\left(\rho_{pi}C_{pi}\right)\frac{\partial T}{\partial t}=\nabla \left(k_{pi}\nabla T\right)$$

For the copper bonded substrate, the subscript ‘cop’ denotes Cu, and the subscript ‘pi’ denotes polyimide. Here, $\rho_{cop}$ denotes the equivalent density of the Cu electrode, $C_{cop}$ denotes the specific heat capacity of the Cu electrode, $\sigma_{cop}$ represents the electrical conductivity of the Cu electrode, T represents the temperature, t represents the time, $k_{cop}$ denotes the thermal conductivity of the Cu electrode, $\rho_{pi}$ denotes the equivalent density of the polyimide, $C_{pi}$ denotes the specific heat capacity of the polyimide, and $k_{pi}$ represents the thermal conductivity of the polyimide.

(12) $$\vec{J}=\sigma \vec{E}$$

The electric current density vector corresponds to the electric field density vector and the electric resistivity. Here, $\vec{J}$ denotes the electric current density vector, $\vec{E}$ denotes the electric field density vector, and σ denotes the electric resistivity.

(13) $$\vec{E}=-\nabla \varphi +S_{p/n}\left(T\right)\nabla T$$

The electric field density vector can be obtained by the electric potential and Seebeck motive force. Here, $\vec{E}$ denotes the electric field density vector, φ denotes the electric potential, $S_{p/n}\left(T\right)\nabla T$ represents the Seebeck motive force.

Because of the electrical current’s continuity properties, the current flowing through the components follows this rule below:

(14) $$\nabla \cdot \vec{J}=0$$

Here, $\vec{J}$ denotes the current density. After setting up the phase-change module and thermoelectric module, we mainly focus on the transient temperature changes and COP of the WTEC.

#### 3.2.3. Assumed Conditions

To simplify this study, we make the following assumptions in the simulation:

1. Three-dimensional transient heat transfer is considered;
2. Negligible contact thermal resistance is assumed;
3. The surface of the PCCM is thermally insulated and isotropic;
4. The ambient temperature is 25 °C, corresponding to room temperature.

#### 3.2.4. Simulation Results

Based on Equation (2), both the PA content and geometric dimensions (thickness) of PCCM significantly influence its thermal capacity. Consequently, we systematically investigated the impact of these parameters on the cooling performance of the TEC system.

**Figure 2:**
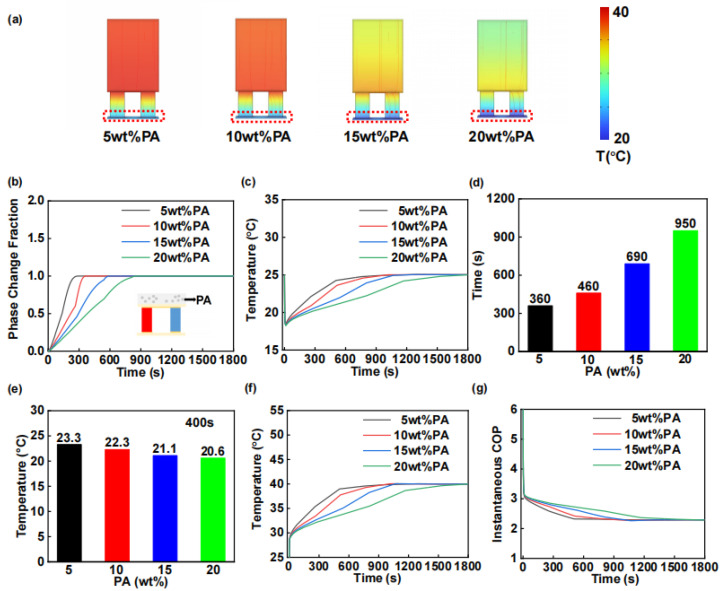
Simulation analysis of the effect of PA content on the cooling performance of WTEC, with PA content from 5 wt% to 20 wt% with 5 wt% interval. (**a**) Temperature cloud of WTEC after 400 s of on-current. (**b**) Phase-change fraction curve. (**c**) WTEC cold surface temperature curve. (**d**) Cooling duration of WTEC. (**e**) Cold surface temperature after 400 s of on-current. (**f**) WTEC hot surface temperature. (**g**) Instantaneous COP of WTEC.

Figure 2a illustrates the temperature distribution of the WTEC system following 400 s of electrical current application, revealing an inverse relationship between PA content and the hot end temperature. Figure 2b depicts the temporal evolution of the phase-change percentage in the PCCM. The complete phase-change durations for PCM mass fractions of 5, 10, 15, and 20 wt% were determined to be 297, 373, 605, and 871 s, respectively. These results demonstrate a direct correlation between PCM concentration and the material’s thermal energy storage capacity. Figure 2c demonstrates the temperature variation profile on the cold side of the WTEC as a function of PA content. The cooling durations (ΔT > 2 °C) are also summarized in Figure 2d. Specifically, the WTEC maintains cooling above 2 °C for 360, 460, 690, and 950 s when the PA content is 5, 10, 15, and 20 wt%, respectively. Following 400 s of current application, the cold surface temperatures were recorded at 23.3, 22.3, 21.1, and 20.6 °C for increasing PA contents, as illustrated in Figure 2e. The collective analysis of Figure 2c–e indicates a significant enhancement in cooling performance with elevated PA content, manifested through both extended cooling duration and reduced operational temperatures.

According to the research literature, the increase in PCM content can directly lead to the larger specific heat capacity of the thermal layer in the phase-transition temperature range [48]. Equation (2) reveals that the thermal capacity at the hot end of the thermoelectric device undergoes significant enhancement during the phase transition process. This phenomenon is clearly demonstrated in Figure 2f, which shows a marked reduction in the temperature rise rate at the hot end during the material’s phase-transition period. Concurrently, the COP was systematically evaluated, as presented in Figure 2g. The results demonstrate a positive correlation between the COP value and increasing PA content. This phenomenon can be attributed to the enhanced heat absorption capacity of the PCCM with increased PA content, which promotes more efficient thermal energy transfer from the hot surface. Consequently, this mechanism effectively reduces the temperature gradient between the hot and cold surfaces.

**Figure 3:**
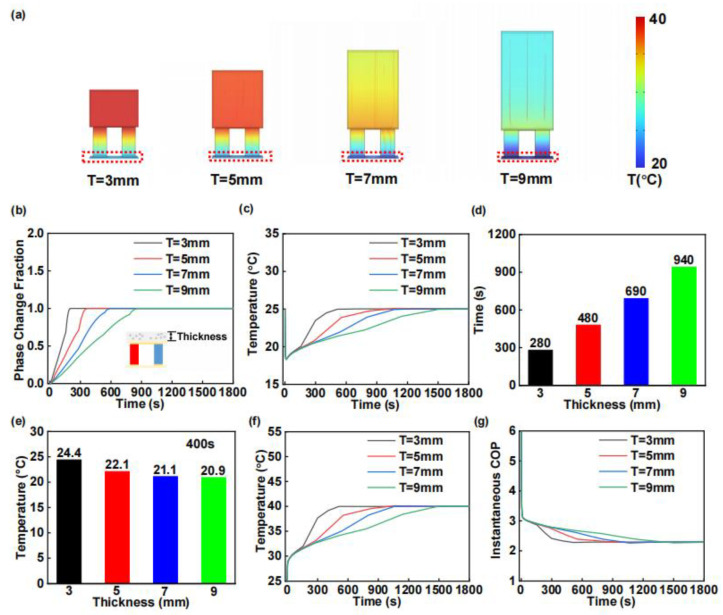
Simulation analysis of the effect of PCCM thickness on the cooling performance of WTEC, with thickness from 3 mm to 9 mm with 2 mm interval. (**a**) Temperature cloud of WTEC after 400 s of on-current. (**b**) Phase-change fraction curve. (**c**) Cold surface temperature curve. (**d**) Cooling duration of WTEC. (**e**) Cold surface temperature after 400 s of on-current. (**f**) Hot surface temperature. (**g**) Instantaneous COP of WTEC.

In addition to the PA content, we also investigated the influence of the geometric dimensions (thickness) of the PCCM on the cooling performance of thermoelectric devices. Figure 3a presents the temperature distribution of the WTEC following 400 s of current application, demonstrating that increased PCCM thickness results in significantly reduced operational temperatures at the thermal surface. Figure 3b depicts the temporal evolution of phase transition percentage within the PCCM material system. The complete phase transition durations were measured to be 206, 376, 605, and 885 s for PCCM thicknesses of 3, 5, 7, and 9 mm, respectively, revealing a positive correlation between PCCM thickness and phase-change completion time. Figure 3c demonstrates a characteristic trend in cold surface temperature variation with increasing PCCM thickness: an initial decrease to a minimum threshold value, followed by a gradual temperature recovery. The experimental results reveal that increased PCCM thickness significantly reduces the cold surface temperature of the WTEC. The cooling durations (ΔT > 2 °C) are also summarized in Figure 3d. Specifically, the WTEC maintains cooling above 2 °C for 280, 480, 690, and 940 s when the PCCM thickness is 3, 5, 7, and 9 mm, respectively. As quantitatively demonstrated in Figure 3e, the cold surface temperatures were recorded at 24.4, 22.1, 21.1, and 20.9 °C, respectively, following 400 s of current application.

As the thickness of the PCCM increases, so does the mass $M_{PCCM}$. Consequently, the rate of temperature increase at the hot end $\frac{\mathrm{d}T_{h}}{\mathrm{d}t}$ decreases during the phase transition of the composite material, as shown in Figure 3f. Utilizing Equation (1) for calculation, Figure 3g illustrates the obtained instantaneous COP, which increases with the thickness of the PCCM during its operation. This phenomenon arises because the increased thickness of the PCCM enhances the overall heat capacity at the hot end and accelerates heat transfer from the hot surface to the PCCM, thereby reducing the temperature difference between the hot and cold surfaces.

### 3.3. Experimental Results

#### 3.3.1. Experimental Verification

**Figure 4:**
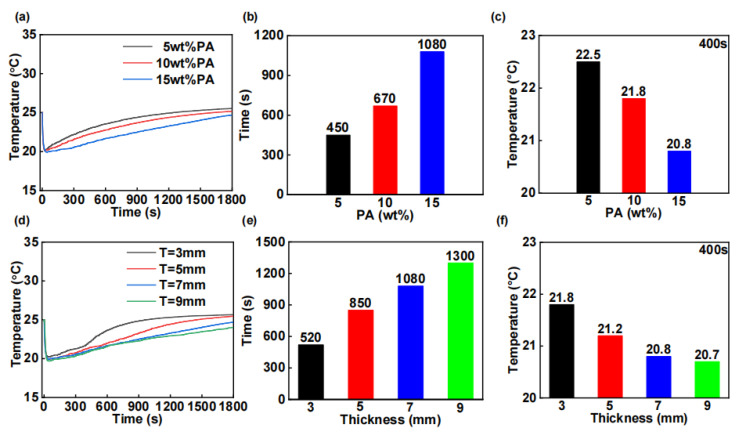
Experimental analysis of the effect of PA content on the cooling performance of WTEC is shown in (**a**–**c**). (**a**) Cold surface temperature of WTEC. (**b**) Cooling duration of WTEC. (**c**) Cold surface temperature after 400 s of on-current. Experimental analysis of the effect of PCCM thickness on the cooling performance of WTEC is shown in (**d**–**f**). (**d**) Cold surface temperature of WTEC. (**e**) Cooling duration of WTEC. (**f**) Cold surface temperature after 400 s of on-current.

Figure 4a–c reveal a significant positive correlation between cooling duration and PA content at a fixed device thickness of 7 mm. Quantitative analysis shows that increasing the PA content from 5 to 15 wt% results in a progressive extension of the cooling period from 450 to 1080 s, with a measured duration of 670 s at the intermediate content of 10 wt%. Furthermore, the minimum achievable temperature shows an inverse relationship with PA content, decreasing progressively with higher PA content.

Additionally, when the PA content is fixed at 15 wt%, increasing the PCCM thickness from 3 mm to 9 mm (in 2 mm increments) leads to longer cooling durations of 520, 850, 1080, and 1300 s, respectively, as shown in Figure 4d–f. These experimental results exhibit the same trends as the simulation outcomes.

It should be noted that a larger geometric thickness will deteriorate the flexibility of the heat dissipation layer. Therefore, considering both flexibility and heat dissipation capacity, we ultimately chose a geometric dimension of 7 mm. In summary, the final selected heat dissipation layer has a PA content of 15 wt% and a geometric thickness of 7 mm.

To enhance thermoelectric response or ensure optimal adaptation of the entire device to the electrical load, we applied varying currents and monitored the resulting variation in the cold surface temperature of the WTEC over time [49]. The findings are presented in Figure S4. It is evident that the cold surface temperature of the WTEC reaches its minimum value at an applied current of 0.30 A. However, the temperature does not decrease further at 0.35 A, as the Joule heating generated by the device begins to exceed the heat absorbed by the Peltier effect [34]. Additionally, the rate of recovery of the cold surface temperature at 0.35 A is faster than that at 0.30 A. Based on these experimental observations, we selected a current of 0.30 A to achieve optimal electrical load.

#### 3.3.2. WTEC and Materials Performance

For body temperature regulation applications, the flexibility and structural stability of the WTEC system represent critical parameters that significantly influence its practical implementation and operational reliability.

**Figure 5:**
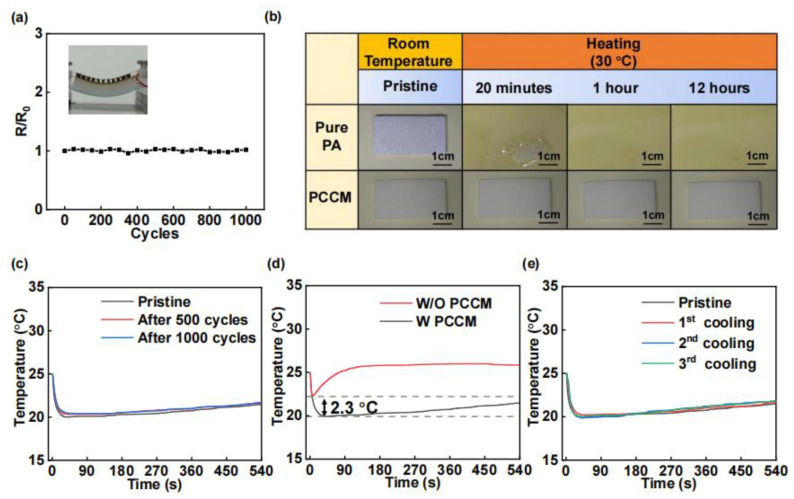
Stability testing of WTEC and PCCM. (**a**) The remarkable stability of resistance during 1000 times of bending of the WTEC. (**b**) Anti-leakage features of PCCM. (**c**) The stability of the cooling effect of the WTEC before bending, after 500 times of bending, and after 1000 times of bending. (**d**) The cold surface temperature of the thermoelectric cooler with and without PCCM. (**e**) The stability of the cooling performance of the WTEC after three prolonged exposures to the melting point temperature and then cooled.

Figure 5a illustrates the WTEC’s flexibility [50], demonstrating its ability to bend with a curvature radius of 40 mm, along with its internal resistance stability after undergoing 1000 repeated bending cycles. Figure 5b highlights the superior anti-leakage properties of the PCCM. The pure PA fully melted within 30 min at a temperature of 30 °C, while the PCCM exhibits no significant leakage, where 30 °C is the melting point of the PA. Figure 5c presents the cooling test results for the WTEC under conditions of no bending and 500 and 1000 bending cycles, indicating a consistent cooling performance across these conditions. Figure 5d contrasts the cold end temperatures of the thermoelectric cooler with and without the PCCM, highlighting that the phase-change heat sink effectively lowers the cold surface temperature of the thermoelectric cooler. The lowest temperature of the cold surface of the WTEC with phase-change heat sink is 2.3 °C lower than that without phase-change heat sink. Figure 5e exhibits the stable cooling performance of the WTEC following three extended exposures to the melting point temperature and cooling, affirming the reusability of the WTEC.

**Figure 6:**
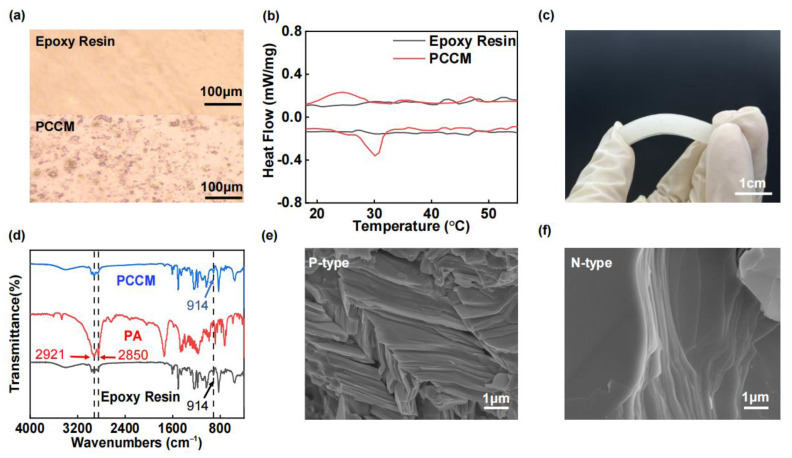
(**a**) Optical microscope photo of Epoxy Resin and PCCM. (**b**) DSC curve of Epoxy Resin and PCCM. (**c**) Optical photo of PCCM. (**d**) FT-IR curve of PCCM, PA, and Epoxy Resin. (**e**) SEM image of p-type thermoelectric leg. (**f**) SEM image of n-type thermoelectric leg.

Optical microscope images of pure Epoxy Resin and PCCM (15 wt%) are shown in Figure 6a, indicating that the PA particles are well encapsulated within the Epoxy Resin. Figure 6b displays the DSC curves [51] of pure Epoxy Resin and the PCCM. The range of heat absorption peaks for the PCCM demonstrates its effectiveness in thermal management within the desired temperature range. Figure 6c depicts an optical photograph of the PCCM, illustrating its good flexibility. FT-IR spectra of pure Epoxy Resin, pure PA, and the PCCM are presented in Figure 6d. The PA curve depicted that there were peaks at 2921 and 2850 cm^−1^,which were characteristic peaks [52]. The curves for both PCCM and Epoxy Resin exhibit a characteristic peak at 910 cm^−1^, which is indicative of the Epoxy Resin. The FT-IR spectra of the PCCM closely resemble those of pure Epoxy Resin due to the low PA content. No new peaks appear in the FT-IR spectrum of the PCCM, indicating that the Epoxy Resin and PA are physically mixed without undergoing chemical reactions [53]. The SEM images of the p-type and n-type thermoelectric legs are presented in Figure 6e and Figure 6f, respectively. It is evident that the layer structure of the n-type material is significantly larger than that of the p-type material. Additionally, the p-type material exhibits lower density compared to the n-type material. These structural differences explain why the thermal conductivity of the n-type material is higher than that of the p-type material.

#### 3.3.3. Device Application

Human beings rely on their skin to perceive external temperatures. In hot environments, lowering the temperature of the skin’s surface can effectively enhance human comfort. Of course, excessively low skin temperature can also cause discomfort.

**Figure 7:**
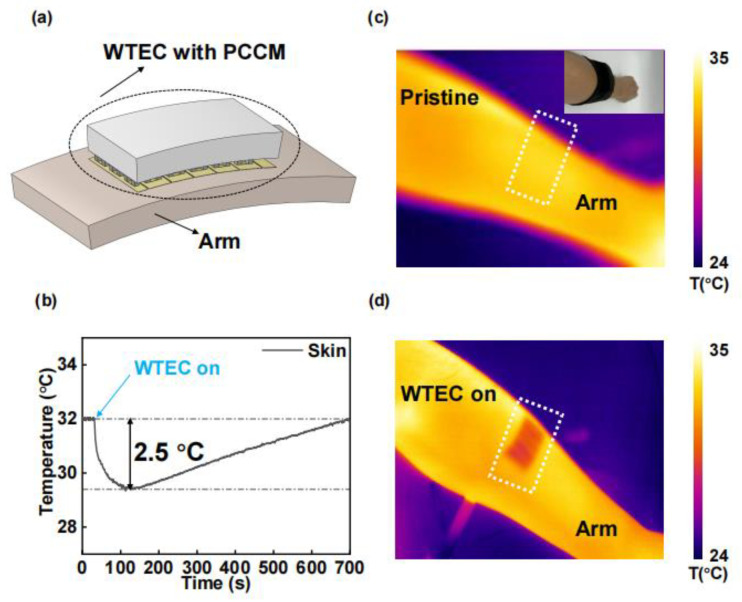
Cooling effect of human skin with WTEC. (**a**) Schematic of WTEC cooling on human arm. (**b**) Skin temperature change curve. Infrared images of arm (**c**) before cooling and (**d**) after cooling.

Given that most of the human skin surface is curved [24], flexible cooling devices better meet the needs of wearable temperature control. Here, we also conducted tests and validation of the wearable cooling characteristics of the WTEC on human skin. Figure 7a illustrates the superior skin adherence of the WTEC due to its flexibility. The on-body temperature when wearing the WTEC was tested in Figure 7b, showing the largest temperature drop of 2.5 °C with a cooling duration of up to 10 min, and its cooling performance was further proved by the infrared images in Figure 7c,d, which were in accord with the WTEC off and on, respectively.

## 4. Conclusions

In this work, we successfully prepared PCCM with both flexibility and the property and it does not leak, which could serve as the heat sink layer for flexible TECs. Through theoretical analysis, simulation, and experimental tests, it was verified that both the PA content and geometry parameters, namely the thickness, could affect the heat capacity of the PCCM heat sink, thereby leading to the cooling performance optimization of the WTEC. The experimental results show that the higher the PA content in the PCCM, the better the cooling effect of the WTEC, and the longest cooling time can reach 1080 s; similarly, the larger the geometry (thickness) of the PCCM, the better the cooling effect of the WTEC, of which the longest cooling time can reach 1080 s when the PCCM is 7 mm. The PCCM was also proved to be anti-leakage even at the transition temperature of 30 °C for at least 12 h. Moreover, the cold surface temperature of the WTEC with PCCM can be further reduced by 2.3 °C compared to the WTEC without PCCM. The WTEC combined with the PCCM heat sink was also fabricated with a proper temperature drop of 2.5 °C and a cooling duration of up to 10 min on human skin. The study reveals an approach that may be used to address long-lasting body temperature regulation in the future.

## Data Availability

The original contributions presented in this study are included in the article/Supplementary Materials. Further inquiries can be directed to the corresponding authors.

## Supplementary Materials

The following supporting information can be downloaded at: https://www.mdpi.com/article/10.3390/ma18071576/s1, Figure S1: Rendering of the FPCB; Figure S2: Fabrication process of phase-change composite material; Figure S3: Preparation process of thermoelectric cooler; Figure S4: Variation in the cold surface temperature of WTEC with time under different applied currents; Table S1: Comparison of this study with other studies about WTEC; Table S2: Thermal conductivity of WTEC and PCCM (303 K).

## Abbreviations

The following abbreviations are used in this manuscript:

WTEC	Wearable thermoelectric cooler
TEC	Thermoelectric cooler
PCCM	Phase-change composite material
COP	Coefficient of performance
PCM	Phase-change material
PA	Paraffin
FPCB	Flexible printed circuit board
DSC	Differential scanning calorimetry
SEM	Scanning electron microscope
N_2_	Nitrogen
FT-IR	Fourier transform infrared spectrometer

## References

[1] Hu R., Liu Y., Shin S., Huang S., Ren X., Shu W., Cheng J., Tao G., Xu W., Chen R. (2020). Emerging Materials and Strategies for Personal Thermal Management. Adv. Energy Mater..

[2] Ma Z., Zhao D., She C., Yang Y., Yang R. (2021). Personal Thermal Management Techniques for Thermal Comfort and Building Energy Saving. Mater. Today Phys..

[3] Veselý M., Zeiler W. (2014). Personalized Conditioning and Its Impact on Thermal Comfort and Energy Performance—A Review. Renew. Sustain. Energy Rev..

[4] Xue S., Huang G., Chen Q., Wang X., Fan J., Shou D. (2024). Personal Thermal Management by Radiative Cooling and Heating. Nano-Micro Lett..

[5] Shoeibi S., Kargarsharifabad H., Sadi M., Arabkoohsar A., Mirjalily S.A.A. (2022). A Review on Using Thermoelectric Cooling, Heating, and Electricity Generators in Solar Energy Applications. Sustain. Energy Technol. Assess..

[6] Chen W.-Y., Shi X.-L., Zou J., Chen Z.-G. (2022). Thermoelectric Coolers: Progress, Challenges, and Opportunities. Small Methods.

[7] Zaferani S.H., Sams M.W., Ghomashchi R., Chen Z.-G. (2021). Thermoelectric Coolers as Thermal Management Systems for Medical Applications: Design, Optimization, and Advancement. Nano Energy.

[8] Jiang L., Zhang H., Li J., Xia P. (2019). Thermal Performance of a Cylindrical Battery Module Impregnated with PCM Composite Based on Thermoelectric Cooling. Energy.

[9] Huo W., Xia Z., Gao Y., Guo R., Huang X. (2023). Flexible Thermoelectric Devices with Flexible Heatsinks of Phase-Change Materials and Stretchable Interconnectors of Semi-Liquid Metals. ACS Appl. Mater. Interfaces.

[10] Chandel R., Chandel S.S., Prasad D., Dwivedi R.P. (2022). Prospects of Sustainable Photovoltaic Powered Thermoelectric Cooling in Zero Energy Buildings: A Review. Int. J. Energy Res..

[11] Sun X., Ling L., Liao S., Chu Y., Fan S., Mo Y. (2018). A Thermoelectric Cooler Coupled with a Gravity-Assisted Heat Pipe: An Analysis from Heat Pipe Perspective. Energy Convers. Manag..

[12] Hyland M., Hunter H., Liu J., Veety E., Vashaee D. (2016). Wearable Thermoelectric Generators for Human Body Heat Harvesting. Appl. Energy.

[13] Cai Y., Wang Y., Liu D., Zhao F.-Y. (2019). Thermoelectric Cooling Technology Applied in the Field of Electronic Devices: Updated Review on the Parametric Investigations and Model Developments. Appl. Therm. Eng..

[14] Guclu T., Cuce E. (2019). Thermoelectric Coolers (TECs): From Theory to Practice. J. Electron. Mater..

[15] Zhao D., Lu X., Fan T., Wu Y.S., Lou L., Wang Q., Fan J., Yang R. (2018). Personal Thermal Management Using Portable Thermoelectrics for Potential Building Energy Saving. Appl. Energy.

[16] Siddique A.R.M., Venkateshwar K., Mahmud S., Van Heyst B. (2020). Performance Analysis of Bismuth-Antimony-Telluride-Selenium Alloy-Based Trapezoidal-Shaped Thermoelectric Pallet for a Cooling Application. Energy Convers. Manag..

[17] Liu X., Zheng F., Fu Q., Song G., Xiong Y. (2024). Thermoelectric Cooler with Embedded Teardrop-Shaped Milli-Channel Heat Sink for Electronics Cooling. Appl. Therm. Eng..

[18] He Z., Yu Q., Ye J., Yan F., Li Y. (2024). Optimization of Plate-Fin Heat Exchanger Performance for Heat Dissipation of Thermoelectric Cooler. Case Stud. Therm. Eng..

[19] Wu B., Lin Y., Tian Y., Wei W., Xu Y., Hu Y., Li J., Li K., Hou C., Zhang Q. (2024). Bioinspired Wearable Thermoelectric Device Constructed with Soft-Rigid Assembly for Personal Thermal Management. Adv. Funct. Mater..

[20] Park H., Lee D., Kim D., Cho H., Eom Y., Hwang J., Kim H., Kim J., Han S., Kim W. (2018). High Power Output from Body Heat Harvesting Based on Flexible Thermoelectric System with Low Thermal Contact Resistance. J. Phys. D Appl. Phys..

[21] Park H., Kim D., Eom Y., Wijethunge D., Hwang J., Kim H., Kim W. (2017). Mat-like Flexible Thermoelectric System Based on Rigid Inorganic Bulk Materials. J. Phys. D Appl. Phys..

[22] Wei H., Zhang J., Han Y., Xu D. (2022). Soft-Covered Wearable Thermoelectric Device for Body Heat Harvesting and on-Skin Cooling. Appl. Energy.

[23] Lund A., Tian Y., Darabi S., Müller C. (2020). A Polymer-Based Textile Thermoelectric Generator for Wearable Energy Harvesting. J. Power Sources.

[24] Hou Y., Yang Y., Wang Z., Li Z., Zhang X., Bethers B., Xiong R., Guo H., Yu H. (2022). Whole Fabric-Assisted Thermoelectric Devices for Wearable Electronics. Adv. Sci..

[25] Prunet G., Pawula F., Fleury G., Cloutet E., Robinson A.J., Hadziioannou G., Pakdel A. (2021). A Review on Conductive Polymers and Their Hybrids for Flexible and Wearable Thermoelectric Applications. Mater. Today Phys..

[26] Heydari Gharahcheshmeh M., Dautel B., Chowdhury K. (2025). Enhanced Carrier Mobility and Thermoelectric Performance by Nanostructure Engineering of PEDOT Thin Films Fabricated via the OCVD Method Using SbCl5 Oxidant. Adv. Funct. Mater..

[27] Shen L., Zhang W., Liu G., Tu Z., Lu Q., Chen H., Huang Q. (2020). Performance Enhancement Investigation of Thermoelectric Cooler with Segmented Configuration. Appl. Therm. Eng..

[28] Liu D., Cai Y., Zhao F.-Y. (2017). Optimal Design of Thermoelectric Cooling System Integrated Heat Pipes for Electric Devices. Energy.

[29] Lyu Y., Siddique A.R.M., Majid S.H., Biglarbegian M., Gadsden S.A., Mahmud S. (2019). Electric Vehicle Battery Thermal Management System with Thermoelectric Cooling. Energy Rep..

[30] Xiang H., An J., Zeng X., Liu X., Li Y., Yang C., Xia X. (2019). Preparation and Properties of Polyurethane Rigid Foam Materials Modified by Microencapsulated Phase Change Materials. Polym. Compos..

[31] Huang Q., Li X., Zhang G., Kan Y., Li C., Deng J., Wang C. (2022). Flexible Composite Phase Change Material with Anti-Leakage and Anti-Vibration Properties for Battery Thermal Management. Appl. Energy.

[32] Bhuiya R., Shah N., Arora D., Krishna N.V., Manikandan S., Selvam C., Lamba R. (2022). Thermal Management of Phase Change Material Integrated Thermoelectric Cooler with Different Heat Sink Geometries. J. Energy Storage.

[33] Alkan C., Sari A. (2008). Fatty Acid/Poly(Methyl Methacrylate) (PMMA) Blends as Form-Stable Phase Change Materials for Latent Heat Thermal Energy Storage. Sol. Energy.

[34] Cheng C.-H., Huang S.-Y., Cheng T.-C. (2010). A Three-Dimensional Theoretical Model for Predicting Transient Thermal Behavior of Thermoelectric Coolers. Int. J. Heat Mass Transf..

[35] Alvarez-Rodriguez M., Alonso-Martinez M., Suarez-Ramon I., José García-Nieto P. (2024). Numerical Model for Determining the Effective Heat Capacity of Macroencapsulated PCM for Building Applications. Appl. Therm. Eng..

[36] Chern B.-C., Moon T.J., Howell J.R., Tan W. (2002). New Experimental Data for Enthalpy of Reaction and Temperature- and Degree-of-Cure-Dependent Specific Heat and Thermal Conductivity of the Hercules 3501-6 Epoxy System. J. Compos. Mater..

[37] Said Z., Pandey A.K., Tiwari A.K., Kalidasan B., Jamil F., Thakur A.K., Tyagi V.V., Sarı A., Ali H.M. (2024). Nano-Enhanced Phase Change Materials: Fundamentals and Applications. Prog. Energy Combust. Sci..

[38] Aftab W., Usman A., Shi J., Yuan K., Qin M., Zou R. (2021). Phase Change Material-Integrated Latent Heat Storage Systems for Sustainable Energy Solutions. Energy Environ. Sci..

[39] Talele V., Moralı U., Najafi Khaboshan H., Patil M.S., Panchal S., Fraser R., Fowler M. (2024). Improving Battery Safety by Utilizing Composite Phase Change Material to Delay the Occurrence of Thermal Runaway Event. Int. Commun. Heat Mass Transf..

[40] Li C., Jin J., Cao W., Sun X., Ding Q., Hou Y., Wang Z. (2024). Finite Element Analysis and Design of a Flexible Thermoelectric Generator with a Rhombus Gap Structure. ACS Appl. Mater. Interfaces.

[41] Wang C., Mobedi M., Yang X., Shen Y., Zhao H., Chen H., Zhang T., Zheng X. (2024). A Comparison Study of Heat Dissipation Module between the Consolidated and Unconsolidated Porous Structures for Thermoelectric Cooler. Appl. Therm. Eng..

[42] Choi J., Dun C., Forsythe C., Gordon M.P., Urban J.J. (2021). Lightweight Wearable Thermoelectric Cooler with Rationally Designed Flexible Heatsink Consisting of Phase-Change Material/Graphite/Silicone Elastomer. J. Mater. Chem. A.

[43] Amberkar T., Mahanwar P. (2023). Thermal Energy Management in Buildings and Constructions with Phase Change Material-Epoxy Composites: A Review. Energy Sources Part A Recovery Util. Environ. Eff..

[44] Baldry M., Timchenko V., Menictas C. (2019). Optimal Design of a Natural Convection Heat Sink for Small Thermoelectric Cooling Modules. Appl. Therm. Eng..

[45] Cao X., Zhang R., Zhang N., Chen L., Li X. (2023). Flexible Composite Phase Change Material with Improved Hydrophobicity and Thermal Conductivity Characters for Thermal Management. J. Energy Storage.

[46] Cai Y., Hong B.-H., Wu W.-X., Wang W.-W., Zhao F.-Y. (2022). Active Cooling Performance of a PCM-Based Thermoelectric Device: Dynamic Characteristics and Parametric Investigations. Energy.

[47] Cong B., Kong Y., Ye Y., Liu R., Du X., Yu L., Jia S., Qu Z., Jiao B. (2023). A Combined Solution of Thermoelectric Coolers and Microchannels for Multi-Chip Heat Dissipation with Precise Temperature Uniformity Control. Appl. Therm. Eng..

[48] Ling Z., Chen J., Xu T., Fang X., Gao X., Zhang Z. (2015). Thermal Conductivity of an Organic Phase Change Material/Expanded Graphite Composite across the Phase Change Temperature Range and a Novel Thermal Conductivity Model. Energy Convers. Manag..

[49] Prete D., Dimaggio E., Demontis V., Zannier V., Rodriguez-Douton M.J., Guazzelli L., Beltram F., Sorba L., Pennelli G., Rossella F. (2021). Electrostatic Control of the Thermoelectric Figure of Merit in Ion-Gated Nanotransistors. Adv. Funct. Mater..

[50] Zhu P., Luo X., Lin X., Qiu Z., Chen R., Wang X., Wang Y., Deng Y., Mao Y. (2023). A Self-Healable, Recyclable, and Flexible Thermoelectric Device for Wearable Energy Harvesting and Personal Thermal Management. Energy Convers. Manag..

[51] Ling Z., Li S., Cai C., Lin S., Fang X., Zhang Z. (2021). Battery Thermal Management Based on Multiscale Encapsulated Inorganic Phase Change Material of High Stability. Appl. Therm. Eng..

[52] Zhao X., Lei K., Wang S., Wang B., Huang L., Zou D. (2023). A Shape-Memory, Room-Temperature Flexible Phase Change Material Based on PA/TPEE/EG for Battery Thermal Management. Chem. Eng. J..

[53] Li M., Guo Q., Nutt S. (2017). Carbon Nanotube/Paraffin/Montmorillonite Composite Phase Change Material for Thermal Energy Storage. Sol. Energy.

