# Supplementary material for: Performance Study of Wearable Thermoelectric Cooler with Phase-Change Composite Heat Sink
Source: Materials (Basel). 2025 Mar 31;18(7):1576. doi: 10.3390/ma18071576 (PMC11990543; doi:10.3390/ma18071576)
Supplement: Supplementary file 1 [file materials-18-01576-s001.zip › materials-3539480-supplementary.pdf]

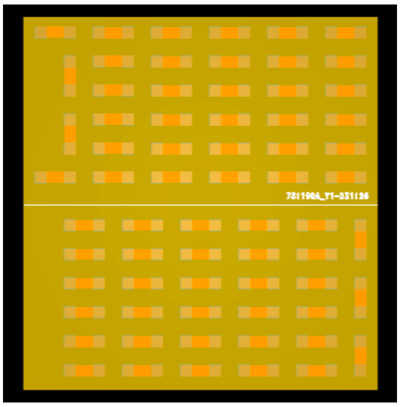

Figure S1. Rendering of the FPCB

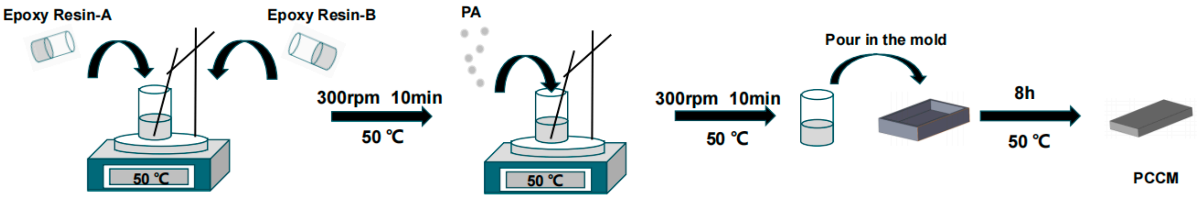

Figure S2. Fabrication process of phase change composite material.

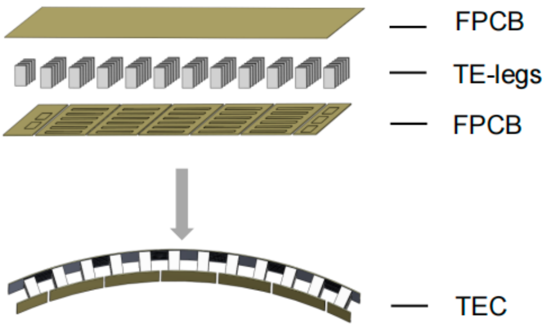

Figure S3. Preparation process of thermoelectric cooler.

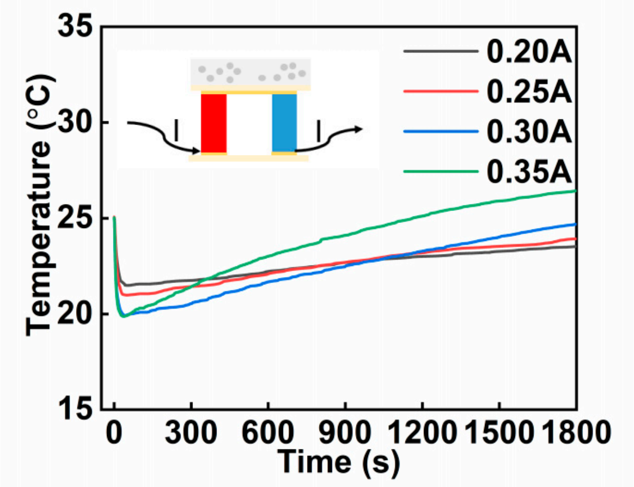

Figure S4. Variation of the cold surface temperature of WTEC with time under different applied currents.

Table S1. Comparison of this study with other studies about WTEC.

Year	theoretical analysis	Simulation studies	Flexible	Time	*ΔT*
2017 [1]	✓	×	✓	-	4 °C
2018 [2]	✓	×	✓	-	4.4 °C
2022 [3]	✓	✓	✓	>10min	1.5 °C
2024 [4]	×	×	✓	2min	1.3 °C
Our work	✓	✓	✓	10min	2.5 °C

Table S2. Thermal conductivity of WTEC and PCCM (303 K).

polyimide	Cu electrode	P-type thermoelectric leg	N-type thermoelectric leg	PCCM
0.113 $W{\cdot}m^{-1}{\cdot}K^{-1}$	396 $W{\cdot}m^{-1}{\cdot}K^{-1}$	1.28 $W{\cdot}m^{-1}{\cdot}K^{-1}$	1.39 $W{\cdot}m^{-1}{\cdot}K^{-1}$	0.203 $W{\cdot}m^{-1}{\cdot}K^{-1}$

References

1. Park, H.; Kim, D.; Eom, Y.; Wijethunge, D.; Hwang, J.; Kim, H.; Kim, W. Mat-like Flexible Thermoelectric System Based on Rigid Inorganic Bulk Materials. *J. Phys. D: Appl. Phys.* **2017**, *50*, 494006. [doi:10.1088/1361-6463/aa94f7]
2. Park, H.; Lee, D.; Kim, D.; Cho, H.; Eom, Y.; Hwang, J.; Kim, H.; Kim, J.; Han, S.; Kim, W. High Power Output from Body Heat Harvesting Based on Flexible Thermoelectric System with Low Thermal

Contact Resistance. *J. Phys. D: Appl. Phys.* **2018**, *51*, 365501. [doi:10.1088/1361-6463/aad270]

3. Wei, H.; Zhang, J.; Han, Y.; Xu, D. Soft-Covered Wearable Thermoelectric Device for Body Heat Harvesting and on-Skin Cooling. *Appl. Energy* **2022**, *326*, 119941. [doi:10.1016/j.apenergy.2022.119941]
4. Wu, B.; Lin, Y.; Tian, Y.; Wei, W.; Xu, Y.; Hu, Y.; Li, J.; Li, K.; Hou, C.; Zhang, Q.; et al. Bioinspired Wearable Thermoelectric Device Constructed with Soft-Rigid Assembly for Personal Thermal Management. *Adv. Funct. Mater.* **2024**, *34*, 2402319. [doi:10.1002/adfm.202402319]
